# The consensus guideline of perioperative antiviral therapy for AIDS patients in China based on clinical practice

**DOI:** 10.3389/fmed.2023.1267236

**Published:** 2023-12-06

**Authors:** Rui Ma, Qiang Zhang, Chang song Zhao, Rugang Zhao, Yao Zhang, Yao shen Zhang, Yong Hai, Guo Wei, Yu Pu, Li qiang Hu, Yanzheng Song, Yu Zhang, Bo yong Hu, Shijie Xin

**Affiliations:** ^1^Department of Orthopedics, Beijing Ditan Hospital, Capital Medical University, Beijing, China; ^2^Department of Orthopaedics, Beijing Chaoyang Hospital, Capital Medical University, Beijing, China; ^3^Public Health and Clinical Center of Chengdu, Chengdu, China; ^4^Department of Otolaryngology, The First Affiliated Hospital of Nanchang University, Nanchang, China; ^5^Shanghai Public Health Clinical Center, Fudan University, Shanghai, China; ^6^Beijing Youan Hospital, Capital Medical University, Beijing, China; ^7^Guangzhou Eighth People’s Hospital, Guangzhou, China; ^8^The First Affiliated Hospital of China Medical University, Shenyang, China

**Keywords:** human immunodeficiency virus, perioperative period, antiretroviral therapy regimen, viral load, expert consensus

## Abstract

The prevalence of human immunodeficiency virus (HIV) and acquired immune deficiency syndrome (AIDS) has emerged as a major public health concern in China. When patients with HIV infection undergo surgical treatment, there are two main challenges. Firstly, medical staff face a high risk of HIV infection due to occupational exposure. Secondly, the patient’s immune function is impaired, increasing the risk of opportunistic infections and postoperative complications. The surgical treatment of such patients is unique, and the risk of occupational exposure during the operation primarily depends upon the viral load of HIV/AIDS patients. Therefore, perioperative antiretroviral therapy is of paramount importance in order to standardize the perioperative antiretroviral therapy (ART) for HIV/AIDS patients. The Surgery Group of the Chinese Association of STD and AIDS Prevention and Control, in collaboration with the Treatment Association, and Surgery Group of the Chinese Medical Association of Tropical Diseases and Parasitology, has developed an expert consensus on perioperative antiretroviral therapy for HIV/AIDS patients. This consensus encompasses various aspects, including surgical risk assessment, selection of perioperative antiretroviral therapy regimens, prevention of opportunistic infections, and the crucial focus on rapid preoperative viral load reduction and immune function reconstruction for HIV/AIDS patients.

## Introduction

The human immunodeficiency virus (HIV) primarily targets human CD4^+^ T lymphocytes, thereby affecting the body’s cellular immune function. Untreated patients will eventually progress to a condition known as acquired immunodeficiency syndrome (AIDS). In 2020, the global count of HIV-infected individuals reached approximately 37.9 million, with an annual increase of around 1.7 million cases. According to the latest data from the China Center for Disease Control and Prevention, by the end of 2020, a total of 1,053,000 HIV/AIDS patients were reported in China. In the same year, 131,671 new cases of HIV infections were reported, along with 351,000 deaths among HIV/AIDS patients. People living with HIV (PLWH) have emerged as a significant public health concern in both China and worldwide (1). With the development of antiretroviral therapy drugs and the widespread adoption of antiretroviral therapy (ART), HIV infection has transformed from a highly lethal disease to a chronic condition that can be effectively managed with medication. The life expectancy of HIV-infected individuals has also approached that of the general population. As PLWH age, an increasing number of them require invasive procedures or surgical operations (2). During surgery, healthcare professionals are primarily concerned about occupational exposure, particularly associated with the high viral load of HIV-positive patients. Therefore, it becomes critical to rapidly reduce the viral load of the surgical patients before the surgery to minimize the risk of occupational exposure. The expert consensus was developed to standardize antiretroviral therapy regimens during the perioperative period. Studies have shown that approximately 25% of people living with HIV (PLWH) will undergo surgical procedures during their lifetime, with 7.8% of them having undergone surgical procedures rated as grade 3 or above (3, 4). Another study revealed that HIV-positive surgical patients primarily undergo four types of surgeries: trauma, infection, tumors, and degenerative dysfunction, accounting for 5.9, 46.9, 23.5, and 23.5%, respectively (5, 6).

The compromised immune function resulting from HIV infection significantly elevates the risk of operative complications during the perioperative period, including surgical site infections, sepsis, and opportunistic infections, among others (7). Therefore, assessing the surgical treatment risk and determining the timing of surgery are of paramount importance. The greatest concern for medical staff during the surgery on HIV/AIDS patients is the risk of occupational exposure. Most doctors are often hesitant to perform surgery on these patients fearing exposure to and a risk of acquiring an HIV infection. In the final analysis, the occurrence of these complications and the risk of occupational exposure are directly linked to the HIV infection and the high viral load of patients. Thus, it is crucial to underscore the administration of rapid and potent ART regimens to lower patient’s viral load before surgery. Studies have demonstrated that ART can significantly reduce the incidence of occupational exposure and the risk of HIV infection, while also minimizing surgical complications (8). To further standardize perioperative antiretroviral therapy regimens for HIV-infected patients, an expert panel has formulated a consensus regimen based on the latest advances in surgical treatment and the findings of clinical and fundamental research (9).

### Preoperative risk assessment and surgical timing

1. General condition assessment of HIV/AIDS patients before surgery: It is paramount to conduct a comprehensive preoperative risk assessment of HIV-positive patients. The main contents include the following:

1. Assess the patient’s general condition: Key factors to consider include the patient’s general well-being and whether they have co-morbidities such as heart disease, hypertension, diabetes, and other important organ diseases.
2. Assessment of the complexity of the patient’s surgery: Assess the complexity of the patient’s procedure and categorize it as either complex or straightforward based on severity. Categories include emergency surgery, limited surgery, or selective surgery.
3. Check the condition of the wound: Assess whether the wound is open or closed and determine the degree of contamination, which can be categorized as Type I incision, Type II incision, or Type III incision.
4. Measuring the CD4^+^ T lymphocyte cell count, viral load, and immune function status of the patients.
5. Evaluating the presence of concurrent infectious diseases such as tuberculosis, syphilis, hepatitis B, hepatitis C, and others.
6. Assess the patient’s nutritional status using common indicators such as albumin, hemoglobin, and body mass index (BMI).

2. The potential surgical risks associated with HIV and opportunistic infection

Research has shown that HIV infection can lead to the reactivation of dormant pathogenic microorganisms in the human body or increase susceptibility to exogenous pathogenic microorganisms, resulting in pathogenic infections (10). HIV-related opportunistic infections primarily involve severe infections (11), which occur due to immunosuppression or suppression of the immune function caused by HIV (12, 13). Biological research on opportunistic infections in HIV-positive patients in China has revealed that *Pneumocystis pneumoniae* (PCP), tuberculosis (TB), and cytomegalovirus (CMV) infections are common opportunistic infections among PLWH.

Common complications of opportunistic infections: A HIV infection complicated by opportunistic infections can definitely necessitate a surgical procedure to remove the infections. For instance, acute CMV infection, mycobacterial infections, and Kaposi’s sarcoma can all result in acute appendicitis (14). Opportunistic infections can also augment surgical risk; for example, pneumocystis and CMV infections increase the risk of cardiovascular disease (15). Tuberculosis and syphilis can elevate serum HIV load, accelerating disease progression after HIV infection and increasing the risk of transmission (16).

Management of common complications of opportunistic infections: PCP should be prevented in patients with CD4^+^ T lymphocyte count <200 cells/μL or a history of oral candidiasis, and a combination of sulfamethoxazole can be used for prevention. For patients who have not received preventive treatment, heightened vigilance should be maintained for PCP when postoperative pulmonary complications arise. Clarithromycin or azithromycin can be chosen for the prevention of *Mycobacterium avium* complex (MAC) infection in AIDS patients with CD4^+^ T lymphocyte count <50 cells/μL (17–19).

While concurrent opportunistic infection remains a leading cause of death in HIV-infected patients, it’s crucial to emphasize that effective antiretroviral therapy can significantly reduce the risk of opportunistic infections by boosting CD4^+^ T lymphocyte counts and restoring immune function. Additionally, some preventive measures against opportunistic infections during the perioperative period can further reduce the risk of infection (20).

3. CD4^+^ T lymphocytes count and surgical timing

The status of immune function is a critical factor to consider in determining surgical requirement and the timing of surgery for HIV/AIDS patients. HIV infection can lead to immune deficiency in the human body, and when the CD4^+^ T lymphocyte count drops below 200 cells/μL, the condition is termed AIDS (21). While current studies indicate that CD4^+^ T lymphocyte count may not significantly determine surgical outcomes in HIV-infected individuals (22), a substantial body of research has demonstrated that a low CD4^+^ T lymphocyte count increases the risk of postoperative complications in HIV/AIDS patients. Research have shown that the incidence of postoperative sepsis in AIDS patients is 48.94%; When the CD4^+^ T lymphocyte count ≤100 cells/μL, the incidence of postoperative sepsis can be as high as 81.25% (23).

In general, when the CD4^+^ T lymphocyte count exceeds 500 cells/μL, HIV infection itself does not significantly impact the feasibility of surgery. HIV-positive patients can undergo the same surgical procedures as non-HIV-infected patients, provided effective protective measures are in place during the operation. When the CD4^+^ T lymphocyte count ranges from 200 to <500 cells/μL, surgical treatment should be approached with caution. It is essential to consider limiting the scope of the operation and minimizing surgical trauma. If the patient has other complications, the surgical treatment plan should be devised with a focus on controlling these complications. When the CD4^+^ T lymphocyte count falls below 200 cells/μL, surgical procedures should be avoided whenever possible. For patients with acute infections, surgery should be delayed or avoided altogether. In cases of emergency surgery, priority should be given to correcting the acute and chronic immune system disorders caused by HIV infection. Major surgery should be minimized, and alternative options such as minimally invasive treatments should be considered, or surgery may be postponed until the patient’s immune function has improved.

If emergency surgery is deemed necessary, a clear communication with patients and their families is essential to explain the benefits, risks, and potential surgical complications. Research has shown that the risk of postoperative complications and death of patients with emergency surgery are significantly increased (24–28).

While assessing the CD4^+^T lymphocytes count, it’s equally important to comprehensively evaluate the organ function and nutritional status of HIV-positive patients. Based on their condition, appropriate drugs, such as thymosin and interferon, can be prescribed to enhance their immune function and administer appropriate treatments for coexisting conditions like hepatitis B, hepatitis C, syphilis, tuberculosis, heart disease, hypertension, and diabetes. Surgical intervention should be considered once the patient’s immune function has significantly improved, and their complications are under control. This approach can help reduce the risk of postoperative complications and mortality.

### Perioperative antiretroviral therapy for HIV/AIDS surgery patients

When patients requiring surgery are found to be positive for both HIV antibody testing and nucleic acid detection of HIV-RNA during preoperative examination, one of the three – emergency surgery, limited surgery, or selective surgery – can be considered based on the patient’s critical condition. If patients require surgery urgently, they can choose integrase strand transfer inhibitors (INSTIs) as part of a rapid and potent antiretroviral therapy regimen. Available medications include Biktatvy (bictegravir, BIC/FTC/TAF) tablets, Genvoya (elvitegravir, E/C/F/TAF) tablets, and Dovato (Lamivudine and Dolutegravir Sodium) tablets. These can also be used in combination with the long-acting fusion inhibitor Albuvirtide (ABT) in the preoperative, intraoperative, and postoperative phases. Opting for a rapid and potent ART regimen before surgery can lead to a swift reduction in the viral load of HIV-positive patients within 2–4 weeks, potentially rendering the virus undetectable in the blood [viral load <20 copies /ml or target not detected (TND)]. This can significantly decrease the risk of surgical site infections and other complications, thereby reducing transmission to healthcare workers after occupational exposure (29–33).

1. Rapid initiation of intensive antiretroviral therapy before surgery and continuous antiretroviral therapy before, during and after surgery

For all newly diagnosed HIV positive patients, World Health Organization (WHO) guidelines recommend initiating ART as soon as possible, ideally within 7 days (34). When considering surgical treatment for HIV-positive patients, it is essential to carefully balance the overall benefits and risks of the timing and regimen of ART in relation to the surgery. Factors to be comprehensively considered include the type of surgery, the urgency of the procedure, the patient’s HIV viral load, CD4^+^T lymphocyte count, and the patient’s economic circumstances. It is advisable to commence rapid and potent ART regimens as early as possible before the surgery. The choice of long-term maintenance antiretroviral therapy after the surgery should take into account the patient’s financial situation.

2. Selection of ART regimens in perioperative period

(1) Selection of ART regimen for surgical patients with HIV infection in the first treatment preoperative period: First, it is advisable to opt for a rapid and potent ART regimen that includes INSTIs, preferably in the form of a single-tablet regimen known for its good compliance. This choice can swiftly reduce the viral load and boost the CD4^+^ T lymphocyte count. Second, the recommended regimen aligns with China’s national free antiretroviral treatment program, which combines two primary drug classes: nucleoside reverse transcriptase inhibitors (NRTIs) and a third drug treatment. For specific details, refer to Table 1 (31).

**Table 1:** Recommended perioperative ART regimens for HIV positive patients.

Regimen	Content of regimen drugs	Combined with drugs
Single tablet regimen (First choice)	BIC/FTC/TAF (Biketarvy, Bictegravir Tablets) Each tablet contains 50 mg Bicrotiravir, 200 mg Emtricitabine, and 25 mg Tenofovir Alafenamide Fumarate	ABT (Albuvirtide for Injection) contains Albuvirtide 160 mg (to be used in combination with Biketarvy or Genvoya during surgery)
	EVG/C/TAF/FTC (Genvoya, Elvitegravir Tablets) Each tablet contained 150 mg of Elvitegravir, 150 mg of Cobicista, 200 mg of Emtricitabine and 10 mg of Tenofovir Alafenamide Fumarate	
Two NRTIs+a third drugs	TAF / FTC (Tenofovir Alafenamide Fumarate / Emtricitabine)	INSTI: DTG, RAL(Integrase inhibitor: Dolutegravir, Raltegravir)
	TDF(ABC^*^) + 3TC(FTC)[Tenofovir dipivoxil fumarate (Abacavir^*^) + Lamivudine (Emtricitabine)]	

HLA*B5701 is recommended to be tested before ABC use to avoid ABC-related hypersensitivity reactions.

(2) Selection of ART regimen for HIV-positive patients: If the HIV viral load is well controlled (viral load <20 copies/ml or TND) and CD4^+^T lymphocyte cell count>200 cells/μL, the decision to either continue the current treatment regimen or transition to a potent and rapidly viral-reducing ART regimen (Table 1) can be made based on the surgical requirements. In the event of viral suppression failure, drug resistance, or allergy reactions, it is crucial to initially assess the patient’s treatment compliance, potential drug interactions, and overall compliance, as adherence plays a pivotal role in the success or failure of the treatment. Ultimately, any decision to change the ART regimen should be made in consultation with health care professionals.

(3) Selection of ART regimen in perioperative fasting and water prohibition before the operation period: If the surgical procedure necessitates fasting and water restriction, oral antiviral medications can be temporarily suspended on the day of surgery. It is advisable to promptly resume oral antiviral drugs following the surgery, particularly for patients with hepatitis B. In general, interrupting oral antiviral drugs for just 1 day is unlikely to compromise their efficacy and effectiveness. In scenarios involving gastrointestinal surgery that require fasting, and oral antiviral drugs cannot be administered, intravenous antiviral drug albuvirtide (ABT) can be considered. After the surgery, the patients should resume oral antiviral drug treatment as soon as possible (35).

(4) Selection of ART regimen for HIV-positive patients with tumors during the perioperative period: Common AIDS-related tumors encompass non-Hodgkin’s lymphoma and Kaposi’s sarcoma. In addition, HIV-positive patients may often present with other organ and system tumors, such as gastrointestinal tumors, liver tumors, lung tumors, and bone and soft tissue tumors. The diagnosis of these tumors primarily relies on pathological biopsies, clinical symptoms, signs, and imaging data. Treatment should be individualized and comprehensive based on the patient’s condition. This may involve a range of modalities, including chemotherapy, immunotherapy, targeted therapy, surgical operation, and radiotherapy (refer to the relevant guidelines for details) (36). Rapid ART initiation is recommended as soon as possible for all HIV-positive patients with cancer. Simultaneously, attention should be given to potential interactions between antiretroviral therapy drugs and antitumor drugs. Whenever feasible, antiretroviral therapy drugs associated with minimal bone marrow suppression and drug interactions should be selected. It is recommended to opt for INSTI-based ART regimens and/or consider combined application with ABT. Encourage a multidisciplinary consultation approach involving experts from multiple departments, including oncology, interventional medicine, infectious disease, and others, to develop a comprehensive diagnosis and treatment plan.

(5) Selection of ART regimen for HIV-positive patients with tuberculosis: The treatment principles for HIV-positive tuberculosis patients are generally akin to those for non-HIV-infected patients. However, special attention should be given to the interaction and potential incompatibility between anti-tuberculosis drugs and antiretroviral therapy drugs during clinical application. Commonly used anti-tuberculosis medications encompass isoniazid, rifampicin, ethambutol, and pyrazinamide. In certain situations, rifapentine, sodium p-aminosalicylate, amikacin, quinolones, and streptomycin may also be considered. For specific dosages, usage instructions, and potential adverse reactions of these drugs, it is advisable to refer to Chinese AIDS diagnosis and treatment guidelines (36, 37). During the perioperative period, careful consideration should be given to ART regimens that exert minimal impact on anti-tuberculosis drugs. Biktatvy (bictegravir, BIC/FTC/TAF) and Genvoya (elvitegravir, E/C/F/TAF) are prudent choices. For patients with tuberculosis, vigilant monitoring of adverse drug reactions and close attention to drug–drug interactions are imperative. Adjustments to the doses of antiretroviral therapy drugs or anti-tuberculosis drugs may be necessary at any time. Consultation with specialists in tuberculosis and infectious diseases is recommended to guide relevant treatment.

### Perioperative prophylactic use of antibiotics

Research has shown that the occurrence of complications like surgical site infections in HIV-positive patients is significantly higher than in HIV-negative patients, primarily due to their compromised immune function. Therefore, the perioperative prophylactic use of antibiotics has to be based on unique considerations. For patients undergoing extensive surgical procedures requiring prolonged operation times, at advanced age and with underlying health conditions, the duration of antibiotic use should be appropriately extended, and the choice of antibiotics should be of a higher grade (38). It is essential to underscore the importance of prophylactic antibiotic use in patients with high-risk factors for surgical site infections. When the CD4 cell count <200 cells/μL, sulfamethoxazole and antifungal drugs can be appropriately used for the prevention and treatment of pneumocystis pneumonia and other fungal infectious diseases (35).

### Possible adverse drug reactions of antiretroviral therapy during perioperative period

The adverse effects of antiretroviral therapy drugs on body organs or tissues are shown in Table 2 (39). Among the common adverse effects associated with INSTIs such as Bictegravir (BIC/FTC/TAF) tablets, nausea, diarrhea, and headache are the most commonly observed. Dolutegravir (DTG) tablets have been associated with common adverse reactions such as insomnia, headache, dizziness, abnormal dream, depression, as well as psychiatric and neurologic symptoms and gastrointestinal symptoms. The common adverse reactions of the long-acting fusion inhibitor (FIs) Albuvirtide (ABT) are diarrhea, headache, dizziness, and rash.

**Table 2:** Adverse effect of antiretroviral therapy regimens drugs on organ/tissues of patients.

Adverse Reactions	Common antiretroviral therapy regimens drugs			
	Nucleoside Reverse Transcriptase Inhibitors (NRTIs)	Non-nucleoside Reverse Transcriptase Inhibitors (NNRTIs)	Protease inhibitors (PIs)	Integrase Inhibitor (INIs)
Cardiovascular disease	ABC: Some cohort studies have been associated with an increased risk of myocardial infarction, with the absolute risk being greatest in patients with traditional cardiovascular risk factors	—	DRV and LPV/r: Some cohort studies have shown an association with cardiovascular events	—
Cardiac conduction disorder	—	RPV, EFV: Prolonged cardiac QT interval	ATV/r, LPV/r: Prolonged cardiac PR interval	—
Hepatotoxicity	AZT: Hepatic steatosis	EFV and NVP: Not recommended for patients with liver insufficiency	All PIs drugs: Drug-induced hepatitis and hepatic decompensation have been reported	BIC/FTC/TAF and EVG/C/TAF/FTC are not recommended for patients with severe liver function damage
Nephrotoxicity / urolithiasis	TDF: Elevated serum creatinine, proteinuria, hypophosphatemia, urinary phosphate loss, diabetes, hypokalemia and non-anionic interstitial metabolic acidosis.	RPV: Inhibit urinary creatinine secretion but does not decrease glomerular function	ATV, LPV/r: Large cohort studies have shown an increased risk of chronic kidney disease	BIC/FTC/TAF and EVG/C/TAF/FTC are Inhibit urinary creatinine secretion without reducing glomerular function; For patients with CrCI estimates <30 mL/min, should not be used
Hypersensitivity reaction	ABC:It is forbidden for HLA-B*5701 positive patients. If ABC allergic reaction is suspected, no matter HLA-B*5701 status, should stop using	NVP: Women are at higher risk than men	—	RAL:Allergic reactions have been reported with RAL in combination with other drugs known to cause allergic reactions; DTG: It is reported in <1% of patients in clinical development projects
Myelosuppression	AZT: Anemia, neutropenia	—	—	—
Allergic reaction	—	NVP > EFV, ETR, RPV: Steven-Johnson syndrome	DRV,ATV/r,LPV/r	RAL: Steven-Johnson syndrome
Myopathy/ Elevated creatine phosphokinase	AZT: Myopathy	—	—	RAL and DTG: Creatine phosphokinase is elevated, and rhabdomyolysis and myopathy or myositis have been reported.
Bone density (BMD)	TDF: BMD loss was more serious than other NRTIs, and it was more significant when combined with synergist. Osteomalacia may be associated with renal tubulopathy and urinary phosphate loss	—	—	—

Abbreviation of antiretroviral therapy regimen:nucleoside analogue reverse transcriptase inhibitors(NRTIs), non-nucleoside reverse transcriptase inhibitors(NNRTIs), protease inhibitors(PIs), integrase strand transfer inhibitors(INSTIs), Abacavir (ABC), Darunavir, (DRV), Lopinave/litonawe (LPV/r), Rilpivrine (RPV), Efavirenz (EFV), Atazanavir (ATV/r), Biketarvy(BIC/FTC/TAF), Genvoya (EVG/C/TAF/FTC), Zidovudine (AZT), Nevirapine (NVP), Dolutegravir (DTG), Bictegravir (BIC), Raltegravir (RAL), Tenofovir disoproxil fumarate (TDF).

## Data availability statement

The original contributions presented in the study are included in the article/supplementary material, further inquiries can be directed to the corresponding author.

## Ethics statement

The manuscript presents research on animals that do not require ethical approval for their study.

## Author contributions

RM: Conceptualization, Methodology, Resources, Supervision, Writing – original draft, Writing – review & editing. QZ: Conceptualization, Writing – review & editing. CZ: Methodology, Writing – review & editing. RZ: Writing – review & editing. YZ: Writing – review & editing, Methodology. YaosZ: Methodology, Writing – review & editing. YH: Methodology, Writing – review & editing. GW: Resources, Writing – review & editing. YP: Resources, Writing – review & editing. LH: Conceptualization, Writing – review & editing. YS: Methodology, Writing – review & editing. YuZ: Methodology, Writing – review & editing. BH: Resources, Writing – review & editing. SX: Resources, Writing – review & editing.
